# NK cell count and glucotransporter 4 (GLUT4) expression in subjects with type 2 diabetes and colon cancer

**DOI:** 10.1186/s13098-016-0152-6

**Published:** 2016-06-13

**Authors:** Paweł Piątkiewicz, Małgorzata Bernat-Karpińska, Tomasz Miłek, Michał Rabijewski, Elżbieta Rosiak

**Affiliations:** Department of Internal Diseases, Diabetology and Endocrinology, Warsaw Medical University, Ul. Kondratowicza 8, 03-242 Warsaw, Poland; Department of General and Vascular Surgery, Warsaw Medical University, Warsaw, Poland; Department of Nuclear Medicine, Warsaw Medical University, Warsaw, Poland

**Keywords:** Diabetes type 2, Colon cancer, GLUT4, NK cells

## Abstract

**Background:**

Type 2 diabetes (T2D) and colon cancer (CC) are numbered among the most common diseases in the world. The decreased activity of natural killer (NK) cells previously revealed in both mentioned pathological states may be correlated with impaired expression of GLUT4 as the major insulin-dependent glucose transporter in these cells.

**Methods:**

The aim of this study was to evaluate GLUT4 expression and NK cells number in subjects with T2D and/or CC in comparison with control group. We evaluated 78 individuals divided into four groups: (1) patients with CC and T2DM, (2) patients with CC, (3) patients with T2DM (4) healthy control. GLUT4 expression on the surface of NK cells was measured using flow cytometry and phenotyping of NK cell was performed by immunofluorescent method.

**Results:**

Subjects with diabetes had the highest GLUT4 expression (21.35 ± 7.2 %) in comparison with other groups (*P* < 0.01). The mean values of GLUT4 expression in group with CC and in patients with both T2D and CC were similar (1.4 ± 0.4 % vs 1.5 ± 1.0 %; respectively). These values were significantly lower than in control group (12.6 ± 2.9 %; *P* < 0.01). In patients with T2D and CC the number of NK cells (20.15 ± 6.6 %) was significantly higher than in other groups, i.e. in group with T2D (14.08 ± 5.7 %), in group with CC (9.21 ± 3.6 %) and in control group (9.48 ± 4.7 %), respectively (*P* < 0.01).

**Conclusions:**

It seems that there is a need to pay more attention to the high incidence of colon cancer among patients with type 2 diabetes. Decreased GLUT4 expression observed on NK cells in patients with colon cancer may be responsible for dysfunction of these cells and the higher carcinogenic risk in type 2 diabetic subjects.

## Background

Type 2 diabetes and colon cancer are currently numbered among the most common diseases in the world. In both diseases, there is a rise in the number of new cases each year and prognosis for upcoming years is unfortunately very worrying. Poland had the highest death rate from colon cancer for both genders in the year 2013—25 % higher in men and 13 % higher in women when compared to the rest of the European Union [1, 2].

Colon cancer is more common in diabetic patients—type 2 diabetes increases the risk of colon cancer about 40–60 % [3–6]. It is certainly a complex relationship which is connected with a sedentary lifestyle, obesity, improper diet, food portions with high glycemic index, altered concentration of endogenous hormones, as well as the presence of hyperinsulinemia, hyperglycemia and insulin resistance that promote tumor growth [7–9].

Glucose is a major energetic substrate for human cells but cellular membranes are impenetrable for glucose, so the cellular transport is possible due to special proteins. There are two types of sugar transporters depending on their use of energy for glucose transport: Na+-dependent glucose co-transporters (SGLT), which require energy for transport, and facilitative Na+-independent sugar transporters (GLUTs), which utilize glucose concentration gradient to move it through membranes, so their action is based on the diffusion [10].

GLUT proteins are a family of particular isoforms which differ from each other in the amino-acidic sequences, but the general outline of their construction is similar—about 50 % of the protein mass is constituted by a highly conservative intracellular domain, the segments of which create 12 a-helices formed into a hydrophilic channel allowing the sugar transport to take place. Other domains are less conservative—extracellular and cytoplasmatic, which contains the N-terminus fragment, C-terminus fragment and the large loop linking segments 6 and 7. Class I of glucotransporters includes isoforms—GLUT1–4 [11–13]. Expression of GLUT isoforms is specific for cells and tissues and modified by hormonal and environmental factors [14]. In previous publications, the author has proved the presence of GLUT-1, GLUT-3, GLUT-4 glucotransporters on the surface of peripheral blood lymphocytes.

In physiological conditions, a quantitative coordination between the activity of molecular intracellular glucose transport system and glucose plasma metabolism exists but in T2D this coordination becomes impaired. Cancer cells are well known to display an enhanced glucose uptake and consumption, and in these cells glucose transporters are dysregulated and they incorporate higher amounts of glucose than normal cells because they need large amounts of energy for their extremely intensive growth and proliferation. Special glucose transporter proteins (GLUTs) are necessary for that process [15]. Many cancer cell lines exhibit an excessive expression of GLUTs [16]. GLUT4 plays a crucial role in glucose metabolism and represents 90 % of glucose transporters [17]. GLUT4 displays an interesting connection with cancer because this transporter is transcriptionally repressed by p53 [18], a tumor suppressor protein important for cell cycle control and apoptosis, processes that are altered usually in cancer.

NK (natural killer) cells that constitute from 5 to 19 % of the peripheral blood lymphocytes are first line of defense against tumor cells. They are characterized by the ability to destroy target cells spontaneously, without prior immunization [19]. It was observed, that the lower NK cell activity is associated with a higher risk of developing cancer [20]. The NK cell number and activity in cancer patients decreases with increasing severity of the cancer [21]. Altered NK cell activity also was shown in obese healthy humans. Laue et al. have demonstrated that NK cells in obese subjects had functional deficits and altered responses after in vitro leptin challenge [22]. Similar disturbances in the functioning of NK cell activity were observed in patients with type 2 diabetes. Disadvantageous alterations of NK cells may lead to impairment in their cytotoxic activity, which can be involved in the increased carcinogenic risk [23], and may contribute in an increased risk of cancer in these patients [24].

Identification of mechanisms underlying the occurrence of colon cancer in patients with type 2 diabetes is important due to the high and steadily increasing incidence of both mentioned diseases in population. So, the aim of this study was to evaluate GLUT4 expression and NK cells number in patients with colon cancer and coexisting type 2 diabetes in comparison with diabetic subjects without cancer, subjects with colon cancer and normal glucose tolerance, as well as with healthy control group.

## Methods

This study was performed in Department of Internal Diseases, Diabetology and Endocrinology in cooperation with Department of General and Vascular Surgery and department of Nuclear Medicine of Warsaw Medical University. Recruitment and qualification of patients was based on general internal examination, with particular emphasis on family interviews and biochemical tests. All people participating in the study were informed in detail about the conducted experiments and the safety principles before they were allowed to give their consent to participate in the study. Qualifying tests were conducted under the protocol approved by the Commission of Bioethics at the Medical University of Warsaw (No. 189/2009 of 20 October 2009).

Patients were divided into four groups: (1) patients with CC in stage T1-4N0M0 and T2DM treated with diet and/or oral antidiabetic agents—(DC), (2) patients with CC in stage T1-4N0M0 with normal glucose tolerance—(C), (3) patients with T2DM treated with diet and/or oral antidiabetic agents without CC and with a negative history of cancer—(D), and (4) healthy individuals who constituted the control group—(control). We excluded patients with renal insufficiency (serum creatinine >1.3 mg/dl), liver diseases, symptomatic heart failure, diabetic complications, using diabetogenic drugs (systemic corticosteroids, oral contraceptives, thiazide diuretics), abusing drugs or alcohol.

The diagnosis of CC was established on the basis of medical history as well as physical and endoscopic examination. Computed tomography of the abdomen was performed to determine the staging of cancer (TNM). The study included patients with clinical diagnosis of CC without lymph node metastases and distant metastases (T1-4N0M0). The operation was conducted within a period of up to 14 days after establishing the clinical diagnosis. Histopathological results verified the clinical diagnosis—in all patients the presence of adenocarcinomas was registered. Due to finding metastasis in mesenteric lymph nodes, four patients were excluded from the C group and four patients from the DC group.

Finally, the analysis included a total of 78 subjects: 19 patients with CC (T1-4N0M0) and T2DM: DC group, 19 patients with CC (T1-4N0M0) and normal glucose tolerance: C group, 20 patients with T2DM: D group, and 20 healthy control group.

### Biochemical tests

We measured fasting plasma glucose (FPG)—with enzymatic method using BIOSEN 5040 analyzer (EKF-Diagnostic GmbH, Germany), glycated hemoglobin (HbA1c)—with high-performance liquid chromatography (HPLC) method using Variant analyzer (Bio-Rad Laboratories Inc, USA), insulin—with radioimmunoassay IMMULITE 2000 Insulin (Siemens Healthcare Diagnostics, USA). Insulin resistance index (HOMA-IR) was calculated (HOMA-IR = fasting insulin (mU/l) × fasting glucose (mmol/l)/22.5), and oral glucose tolerance test (OGTT) with 75 g of glucose was performed according to World Health Organization (WHO) procedures (in groups DC and D).

### GLUT4 expression measurements

GLUT4 expression measurements were performed using flow cytometry method [25]. Mononuclear cells were isolated from blood samples on Gradisol L (fluid density of 1.077 g/L, AQUA-MEDICA) and washed twice in 0.9 % NaCl. To mark the population of cells that do present GLUT4 protein expression monoclonal antibody (MoAb) anti-GLUT4 was used together with single color, indirect immunofluorescence technique. Per single staining process the sample of 106 mononuclear cells was taken. Cells were incubated for 30 min with 2 µL of anti-GLUT4 antibody in an ice bath of 4 °C and next washed with phosphate-buffered solution (PBS) with 0,01 % sodium azide (NaN3). The cells were next incubated for 20 min (ice bath, temp. 4 °C) with 10 µL of secondary, non-specific antibody binding to the immunoglobulin fragment F(ab’)2 and conjugated with fluorochrome-fluorescein isothiocyanate (FITC). Later the mononuclear (MNC) cells were washed again with PBS with 0.01 % NaN3 and suspended in 500 µL of FACS flow (Becton–Dickinson; USA). For data acquisition and analysis the Facs Calibur flow cytometer (Becton–Dickinson; USA) with CellQuest software (Becton–Dickinson; USA) was used. The results were given as the percentage of cells presenting the expression of the investigated protein.

### Phenotyping NK cells

Immunofluorescent phenotyping of NK (CD16^+^) and cytotoxic T cells (CD3^+^CD56^+^) in peripheral blood lymphocytes was performed using specific murine anti-human CD3/CD56 + CD16 FITC-PE-conjugated monoclonal antibodies (Simultest, Becton–Dickinson; USA). Fluorescence analysis was performed using FACS Calibur flow cytometer and the Cellquest program. Lymphocytes T (CD3^+)^ cells were identified with strong green fluorescence, NK cells (CD16^+^) with red fluorescence and cytotoxic lymphocytes (CD3^+^CD56^+^) were identified with green and red fluorescence. These studies were conducted at the Department of Nuclear Medicine.

### Statistical methods

The data were presented as a mean and standard deviation. Quantitative parameters were compared using the Aspin-Welch test with a marginal level of statistical significance equaling *P* = 0.01. Variations in glucose uptake in peripheral blood lymphocytes were analyzed with Student’s test. Multiple factor regression was performed to analyze correlations of NK cells number and GLUT4 expression with all studied clinical parameters.

## Results

The characteristics of the studied groups are shown in Table 1. The duration of diabetes in D group was similar (statistically insignificant) to its duration in DC group. The studied groups did not differ in a statistically significant manner in terms of number, gender, age, BMI and WHR. We noticed significant differences in biochemical parameters between patients in DC and D groups in comparison with the control group. In patients in D group we observed a significantly higher level of FPG (7.24 vs 5.34 mmol/L, *P* < 0.01) and HbA1c (7.23 vs 5.4 %, *P* < 0.01) in comparison to the control group, as well as significantly higher levels of insulin resistance—HOMA-IR: 4.46 vs 1.89 and fasting insulin concentrations: 14.24 vs 7.79 mU/L, respectively (*P* < 0.01).

**Table 1:** Characteristics of the study groups (x ± SD)

Parameter	Control group	D	C	DC
No. pts.	20	20	19	19
Sex, M/F	10/10	10/10	10/9	9/10
Age, years	55.5 ± 8.1	55.4 ± 7.8	56.4 ± 8.0	56.0 ± 8.0
BMI, kg/m^2^	26.6 ± 2.9	27.0 ± 5.1	25.9 ± 3.3	26.3 ± 2.9
WHR, m/m	0.88 ± 0.08	0.89 ± 0.08	0.88 ± 0.09	0.87 ± 0.08
T2D, years	−	6.5 ± 3.1	−	6.7 ± 3.1
FPG, mmol/l	5.3 ± 0.7	7.2 ± 0.8*	5.5 ± 0.8	7.5 ± 0.9*
HbA1c, %	5.4 ± 0.8	7.2 ± 0.7*	5.5 ± 0.7	7.3 ± 0.9*
Insulin, mU/l	7.8 ± 3.5	14.2 ± 1.6*	8.2 ± 4.6	21.7 ± 8.1*
HOMA-IR^a^	1.89 ± 1.05	4.46 ± 1.8*	1.99 ± 1.2	6.96 ± 2.8*

*BMI* body mass index, *WHR* waist/hip ratio, *SBP* systolic blood pressure, *DBP* diastolic blood pressure, *FPG* fasting plasma glucose

Comparing the parameters of various study groups was made relative to the control group; * *P* < 0.01

^a^HOMA-IR index was calculated on the basis of HOMA formula = fasting insulin (mU/l) × fasting glucose (mmol/l)/22.5

In patients in DC group, the mean FPG levels (7.52 vs 5.34 mmol/L, *P* < 0.01) and HbA1c (7.27 vs 5.4 %, *P* < 0.01) were significantly higher than observed in the control group, but did not differ significantly with respect to D group. In addition, patients in DC group were characterized by the highest mean fasting insulin (21.65 mU/L) and the highest value of HOMA-IR (6.96) when compared with different groups of patients, and the differences were statistically significant (*P* < 0.01) In patients in C group, the mean values of the tested biochemical parameters were significantly lower than in patients in D group and also in the DC group. This group did not show statistically significant differences relating the control group (Table 1).

Subjects in DC group differed significantly from those in D group in terms of diabetic therapy. Metformin was the predominant anti-diabetic drug in D group, as it was used in 17/20 patients (85 %). Sulfonylureas were used in 4/20 patients (20 %), acarbose in 3/20 (15 %) and incretin mimetics in 2/20 patients (10 %). The vast majority of patients in DC group (15/19–79 %) was treated with a sulfonylurea, 9/19 (47 %) with metformin, 2/19 (10.5 %) with acarbose and 1/19 (5 %) with incretin mimetics.

### GLUT4 expression

**Fig. 1:**
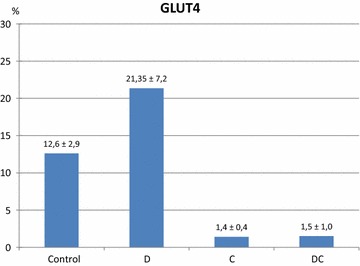
Comparison of the quantitative expression of GLUT4 on the surface of NK cells in the control group, patients with diabetes type 2 (*D*), colon cancer (*C*) and patients with both D and C (*DC*). The differences between study groups are statistically significant (*P* < 0.01)

Patients in D group were characterized by the highest GLUT4 expression on the surface of NK cells in comparison with others groups (21.35 ± 7.2 %), and these differences were statistically significant (*P* < 0.01). This study also showed that the mean values of GLUT4 expression in C group were similar to the mean GLUT4 expression in DC group (1.4 ± 0.4 % vs 1.5 ± 1.0 %; respectively). These values were statistically significantly lower than in control group (12.6 ± 2.9 %; *P* = 0.01) (Fig. 1). We have found no simple correlations between GLUT4 expression and other clinical factors such as age, BMI, WHR, FPG, HOMA, Insulin and HbA1c level in the studied groups (Table 2).

**Table 2:** The correlations between GLUT4 expression and other clinical factors in study groups

Variable	Control		D		C		DC	
	*r*	*P*	*r*	*P*	*r*	*P*	*r*	*P*
GLUT 4 and age	0.56	0.08 NS	0.43	0.06 NS	0.35	0.07 NS	0.36	0.12′ NS
GLUT 4 and BMI	0.43	0.06 NS	0.52	0.11 NS	0.46	0.06 NS	0.52	0.18 NS
GLUT 4 and WHR	0.15	0.55 NS	0.19	0.45 NS	0.22	0.46 NS	0.16	0.39 NS
GLUT 4 and FPG	0.36	0.28 NS	0.39	0.31 NS	0.38	0.37 NS	0.42	0.39 NS
GLUT 4 and HbA1c	0.39	0.19 NS	0.43	0.21 NS	0.39	0.41 NS	0.40	0.45 NS
GLUT 4 and insulin	0.15	0.51 NS	0.16	0.52 NS	0.18	0.18 NS	0.15	0.23 NS
GLUT 4 and HOMA	0.19	0.61 NS	0.22	0.48 NS	0.58	0.22 NS	0.62	0.31 NS

*NS* statistically not significant (*P* < 0.05), *r* Pearson correlation, *D* diabetes mellitus type 2, *C* colon cancer, *DC* diabetes mellitus type 2 and colon cancer

### Number of NK cells

**Fig. 2:**
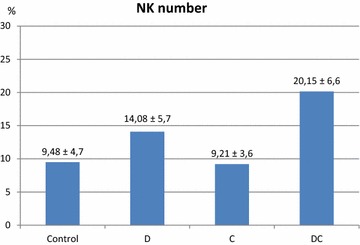
Comparison of the percentage of NK cells in the control group, patients with diabetes type 2 (*D*), colon cancer (*C*) and patients with both *D* and C (*DC*). The differences between study groups are statistically significant (*P* < 0.01)

In D group we noticed a significantly increased number of NK cells in comparison to the control group (14.08 ± 5.7 % vs 9.48 ± 4.7 %, *P* < 0.01). In patients in DC group we observed, that NK cell count was significantly higher in comparison with the control group (20.15 ± 6.6 % vs 9.48 ± 4.7 %, *P* < 0.01). In patients with CC without carbohydrate metabolism disturbances similar number of NK cells in relation to the control group was noted (9.21 ± 3.6 %, ns) (Fig. 2).

Patients in DC group were characterized by a significantly higher NK cell count compared to those from D group (*P* < 0.01), as well as to individuals from C group (*P* < 0.01). In D group, a higher number of NK cells was observed when compared with the C group (*P* < 0.01) (Fig. 2). We have found no correlations between NK cells number and other clinical factors in the studied groups (Table. 3).

**Table 3:** The correlations between NK cells numbers and other clinical factors in study groups

Variable	Control		D		C		DC	
	*r*	*P*	*r*	*P*	*r*	*P*	*r*	*P*
NK number and age	0.21	0.41 NS	0.41	0.07 NS	0.25	0.21 NS	0.46	0.22 NS
NK number and BMI	0.15	0.45 NS	0.42	0.21 NS	0.47	0.09 NS	0.48	0.21 NS
NK number and WHR	0.43	0.52 NS	0.23	0.46 NS	0.34	0.34 NS	0.52	0.32 NS
NK number and FPG	0.18	0.27 NS	0.24	0.30 NS	0.47	0.34 NS	0.32	0.32 NS
NK number and HbA1c	0.23	0.45 NS	0.33	0.29 NS	0.67	0.34 NS	0.29	0.14 NS
NK number and insulin	0.46	0.41 NS	0.26	0.52 NS	0.12	0.34 NS	0.31	0.13 NS
NK number and HOMA	0.42	0.51 NS	0.51	0.43 NS	0.34	0.21 NS	0.58	0.31 NS

*NS* statistically not significant (*P* < 0.05), *r* Pearson correlation, *D* diabetes mellitus type 2, *C* colon cancer, *DC* diabetes mellitus type 2 and colon cancer

## Discussion

The growing incidence of type 2 diabetes as well as neoplasms constitutes a major socio-economic problem. The early identification of patients from the groups of increased risk of these diseases allows the introduction of preventive countermeasures what is especially important in Polish population with the high prevalence of type 2 diabetes and colon cancer.

This study was focused on determination of the impact of type 2 diabetes as well as colon cancer on the number of NK cells and expression of GLUT4 on the surface of these cells. We demonstrated, that in patients with type 2 diabetes but without colon cancer GLUT4 expression was higher than in control group but also significantly higher than in patients with type 2 diabetes and concomitant colon cancer. We also noticed, that the mean values of GLUT4 expression in patients with normal glucose tolerance and colon cancer were similar to the mean GLUT4 expression in patients with colon cancer and type 2 diabetes.

GLUT 4 is present on the surface of leukocytes and the regulation of this expression is associated with the functions of particular cells [25, 26]. GLUT4 can facilitate glucose transport, glucosamine and dehydroascorbic acid and its expression is not only restricted to leukocytes but also is presented in tissue with a marked insulin dependent glucose transport like, heart, adipose tissue, and skeletal muscle [27]. When insulin binds to its receptor, GLUT4 is rapidly translocated to plasma membrane, increasing glucose uptake in the cell. Because muscle and fat tissues comprise a large fraction of the body mass, GLUT4 plays a central role in glucose metabolism and represents 90 % of GLUTs [17]. Moreover, GLUT4 expression is regulated in a tissue-specific manner and magnitude of GLUT4 expression is controlled by different factors, such as diet, exercise and insulin [28]. Finally, GLUT4 displays a connection with cancer, as a transporter is transcriptionally repressed by p53, a tumor suppressor protein important for cell cycle control and apoptosis, processes that are usually altered in cancer [18].

Many cancers display high rates of glucose uptake and one of the explanation is the overexpression of GLUT proteins. GLUTs overexpression has been observed in many cancers and expression levels were inversely correlated with prognosis [16]. Elevated levels of glucose uptake are induced by activated ras or src oncogenes which are key elements in the transduction of multiple signaling pathways [29]. In CC cell lines, mutations in KRAS (v-Ki-ras2 Kirsten rat sarcoma viral oncogene homolog) or BRAF (vraf murine sarcoma viral oncogene homolog B1) genes, are able to trigger an overexpression of GLUT1 and an increase of the glucose uptake. Furthermore, the exposition of CC cell lines to low levels of glucose contributes to the development of mutations in KRAS, which give rise to the upregulation of GLUT1 and to an increase in glucose uptake [30].

Deregulated GLUT4 expression has been described in RCC (renal cell carcinoma). The expression of different GLUTs is altered in a histological subtype-specific manner. In conventional clear cell RCC, GLUT1 expression is increased, while the expression of GLUT4, GLUT9, and GLUT12 decreases versus the healthy kidney. In chromophobe RCC, GLUT4 expression is increased, while the expression of GLUT2 and GLUT5 is decreased [31].

GLUTs expression was also studied in a CC biopsies. These study revealed that GLUT1 expression was associated with tumor progression and poorer prognosis [32]. The expression of GLUT1 and Ki-67, as cell proliferation marker, were also analyzed in different zones of CC samples [33]. In central and superficial part of tumors, no significant differences were detected between GLUT1 expression, Ki-67 expression, and clinic-pathological parameters. However, in the deepest invasive site, GLUT1 expression was associated with Ki-67 labeling index. In addition, in patients who underwent curative surgery, the GLUT1 expression at the deepest invasive site was significantly associated with poorer prognosis. Therefore, GLUT1 expression may be used as predictor of poor prognosis in advanced CC.

In our study we demonstrated significant lowering of GLUT4 expression on NK cells in patients with colon cancer not only in group with coexisting type 2 diabetes but also in subjects with normal glucose tolerance. These findings indicate that disturbances of cellular glucose transport may be present in patients with normal glucose homeostasis and cancer. It must be pointed, that there is the relationship between GLUT4 expression on NK cells surface and their functions which is confirmed by low GLUT4 expression on NK cells observed in patients with colon cancer and associated with impaired NK cells cytotoxicity in these patients [23]. The higher GLUT4 expression on NK cells in patients with type 2 diabetes but without cancer is probably associated with compensative mechanism, which is responsible for proper glucose transport to these cells despite insulin resistance and hyperglycemia. Similar findings, confirm higher GLUT4 expression on NK cells with decreased cytotoxic activity in patients with type 2 diabetes, were showed in other our studies [12, 34].

Our findings confirm hypothesis, that pointed above compensative mechanism is significantly impaired in case with coexisting cancer, because in diabetic patients with colon cancer we observed NK cells activity about fourfold lower when compared with diabetic patients without colon cancer [25] and followed by low GLUT4 expression that we demonstrated in this study. These results are hints suggesting that monitoring of GLUT4 expression on NK cells in patients with type 2 diabetes, which indirectly reflects cytotoxic activity of these cells, may be helpful in proper identification of people with increased risk of cancer.

This study was also focused on determining the impact of type 2 diabetes and colon cancer on the number of NK cells. Our study showed that the NK cell count is increased in subjects with type 2 diabetes without cancer as well as in subjects with coexisting type 2 diabetes and colon cancer when compared to healthy subjects. Simultaneously, we observed no statistically significant difference concerning the number of NK cells in patients with colon cancer and normal glucose tolerance in comparison to the control group. In this study, changes in number of NK cells in the patients with type 2 diabetes and a coexisting colon cancer were accompanied by increased levels of fasting insulin and high HOMA-IR value. We can therefore conclude that there is a correlation between the abnormal number of NK cells and the grade of insulin resistance. These alterations are observed in people with type 2 diabetes whose impaired immune system may lead to an impairment in antitumor defense, and are probably related to hyperinsulinemia.

In our cohort patients with type 2 diabetes but without colon cancer differed from those with type 2 diabetes and colon cancer in terms of applied antidiabetic therapy. Metformin was predominantly used in patients with type 2 and without cancer, while the vast majority of patients with type 2 diabetes and coexisting colon cancer was treated with sulfonylurea drugs. It was shown, that metformin intake reduces the risk of colon cancer [35] and the protective effect of metformin appears to result from its inhibitory effect on intracellular transduction mechanisms stimulated by the activation of insulin receptor/insulin like growth factor (IGF) axis [36].On the contrary, treating of diabetes with drugs stimulating insulin release may increase the risk of cancer.

Proper identification of diabetic patients with increased risk of colon cancer is essential in order to implement adequate preventing procedures. According to guidelines of the Polish Society of Gastroenterology, full colonoscopy is suggested to be the preferred screening method of colon cancer screening in Poland and should be performed once every 10 years in people aged over 50 years and in subjects with an increased risk of this cancer (genetic factors, positive family history). None of scientific societies in Poland considers patients with type 2 diabetes as subjects with a higher risk of developing colon cancer and potential candidates to routine colonoscopy, unfortunately. It was documented, that screening colonoscopy results in significant decline in the incidence of colon cancer and mortality [37–39] but in many cases the diagnosis is established too late. Therefore, there has been a discussion concerning the best screening methods for diagnosis of colon cancer. It seems that there is a need to pay more attention to the high incidence of this cancer among type 2 diabetic patients in order to improve diagnostic and therapeutic methods in this particular group of patients.

Nowadays NK cell-based therapeutic strategies and selective glucose transporter-directed therapies comprise promising option that may improve chance of successful anti-tumor treatment not only in colon cancer [40], but in various types of neoplastic disorders [41–44]. Moreover, the pharmacological inhibition of co-transporters SGLT2 is safe and efficient in treating type 2 diabetes. SGLT2 inhibitors (gliflozins) are medications available in many countries and used in medical practice [45]. It has been recently revealed that one of SGLT2 inhibitors—empagliflozin, when added to standard treatment, reduces the risk of cardiovascular events and death from any cause [46]. It may be supposed that GLUT proteins will be the next target for new antidiabetic drugs.

## Conclusions

Impaired GLUT4 expression and altered number of NK cells are probably associated with increased incidence of colon cancer in patients with type 2 diabetes. Further studies in this field are necessary to explain the pathophysiological mechanisms that lead to the frequent incidence of colon cancer in subjects with type 2 diabetes. Patients with diabetes are at risk of developing colorectal cancer, they must undergo screening tests. Diabetic patients education on this problem is also essential.

## Abbreviations

BMI: body mass index
DC: group with type 2 diabetes and colon cancer
CC: colon cancer
FACS: fluorescence-activated cell sorting
FITC: fluorescein isothiocyanate
FPG: fasting plasma glucose
GLUT: glucotransporter
HbA1c: glycated hemoglobin A1c
HOMA-IR: homeostasis model assessment of insulin resistance
HPLC: high-performance liquid chromatography
IGF: insulin like growth factor
MNC: mononuclear
Mo Ab: monoclonal antibody
NaN3: sodium azide
NK: natural killer
OGTT: oral glucose tolerance test
PBS: phosphate-buffered solution
RCC: renal cell carcinoma
SGLT: Na+- dependent glucose co-transporter
TNM: cancer staging system (tumor, nodes, metastasis)
T2D: type 2 diabetes
WHO: World Health Organization
WHR: waist–hip ratio
